# Induced pluripotent stem cells derived cardiomyocytes from Duchenne Muscular Dystrophy patients in vitro

**DOI:** 10.12669/pjms.37.5.3104

**Published:** 2021

**Authors:** Fareeha Faizan Ghori, Mohsin Wahid

**Affiliations:** 1 Fareeha Faizan Ghori, Dow Research Institute of Biotechnology and Biomedical Sciences, Dow University of Health Sciences, Karachi, Pakistan; 2 Mohsin Wahid, Department of Pathology, Dow International Medical College, Dow University of Health Sciences, Karachi, Pakistan. Dow Research Institute of Biotechnology and Biomedical Sciences, Dow University of Health Sciences, Karachi, Pakistan

**Keywords:** Duchenne muscular dystrophy, Human induced pluripotent stem cells, In vitro cardiomyocytes

## Abstract

**Objective:**

This study aimed at the in vitro generation of DMD-cardiomyocytes from patient-specific induced pluripotent stem cells derived from a Pakistani patient for future work on DMD in vitro disease modeling and drug testing for efficacy and toxicity.

**Methods:**

This in vitro experimental study was carried out from December 2018 to January 2019 at Stem Cells and Regenerative Medicine Lab (SCRML) at Dow Research Institute of Biotechnology and Biomedical Sciences (DRIBBS), Dow University of Health Sciences (DUHS) Urine derived DMD-iPSCs were used which had been generated previously from a Pakistani DMD patient who had been selected through non-random purposive sampling. These were differentiated towards cardiomyocytes using Cardiomyocytes Differentiation media having specified growth factors and then the molecular characterization of the differentiated cells was done using immunofluorescence.

**Results:**

Pakistani patient’s DMD-Cardiomyocytes were generated and their identity was confirmed by positive immunofluorescence for the expression of cardiac markers NKX2-5 and TNNT-2.

**Conclusion:**

This study aimed for in vitro generation of DMD cardiomyocytes for future application in disease modeling, new drug testing for efficacy and toxicity, as well as for drug-testing for tailored personalized therapy. To the best of our knowledge, this was the first time DMD-Cardiomyocytes were generated from Pakistani DMD patients using their own induced pluripotent stem cells.

## INTRODUCTION

The lethal X-linked recessive genetic disorder Duchenne Muscular Dystrophy (DMD) affects ~one in 3500-5000 live born males; securing its status as the most frequently occurring and severe muscular dystrophy.^1^ It is caused by a lack of the sub-sarcolemmal protein dystrophin in skeletal and cardiac muscles where it serves to normally connect the intracellular contractile machinery to the extracellular matrix. The resulting sarcolemmal fragility in response to mechanical stress, cytoskeletal disruption and mitochondrial dysfunction, culminates in cell death.^2^ Attempts at regeneration are ineffective and fibrosis occurs; resulting in rapidly progressive muscular degeneration and weakness.

The heart is involved in 25% DMD individuals at six years of age, 59% at 10 years, majority by 18 years and eventually in up to 90%.^1^,^3^ Death usually occurs at a young age in the late 20s, with 40% being due to cardiac failure, making it the leading cause.^3^ The cardiac component is magnified as advances in mechanical ventilation techniques mitigate the impact of the respiratory component which is the other main cause.^1^

Initially, decreased systolic function and sinus tachycardia occur.^4^ Although Dilated Cardiomyopathy is thought typical of DMD, Left Ventricular Dysfunction, myocardial fibrosis, arrhythmias (in 44%), conduction abnormalities in Pukinje fibers, Congestive Heart Failure and cardiac arrest have also been observed.^1^

The dilemma of the DMD patients’ situation is that currently, no easily available and universally applicable cure exists. Corticosteroids are customarily used to slow progression and mitigate severity while Angiotensin Converting Enzyme Inhibitors and β-Blockers provide cardiac support. Novel promising approaches being researched to fill this void include drugs for gene therapy like the stop codon read-through drug Atalauren with accelerated conditional approval in Europe as Translarna, and the exon-skipping drug Eteplirsen with conditional approval in the U.S as Exondys51.^5^,^6^ Other approaches being investigated include gene-editing for correction of mutation with CRISPR/Cas9 or for restoration of the reading frame.^7^

Human cells are better suited for In vitro work than animal cell due to inherent difference in genes and hence morphology and physiology. Thus results from In vitro-studies on human DMD-skeletal muscles have benefited clinical trials in other humans or the same person; but these skeletal muscle therapeutics don’t improve the cardiac morbidity due to differences in physiology, rate of RNA production, mRNA levels and turnover, Dystrophin levels, leakiness of membrane, exon-skipping drug half-life and drug-toxicity.^3^ Thus, similar In vitro studies on DMD-cardiomyocytes are being employed to aid clinical trials targeting DMD-cardiomyocytes.

These offer a ray of hope for Pakistani families struggling with multiple children being afflicted by this disabling and lethal disease, as well as for new mutations with no prior family history. Never the less, these new generation therapies are mutation specific; while mutations causing DMD are of various types and 25-33% are new mutations, so their type and frequency vary geographically.^8^ This means that these therapies, as well as results from DMD studies conducted in other parts of the world, may not be fully relevant or suited to the different mutation profile of the Pakistani patients.

Therefore in vitro patient-specific DMD-cardiomyocyte from Pakistani patients are required for disease-modeling, existing drugs’ efficacy and toxicity screening, and new drug development; before application of these interventions on Pakistani patients.

Acquiring primary cardiomyocytes through biopsy is an invasive risky procedure and these postnatal adult cardiomyocytes do not expand in vitro. Attempts to aid this have been made by scientists using developmental stage cardiac extracellular matrix to provide growth factors and by overexpression of the cell cycle and proliferation regulator Myc in epicardium; while upstream extra-cardiac endocrine control of epicardial factor involved in cardiac development (Insulin-like growth factor 2) has also been demonstrated.^9^-^11^ While researchers continue to unravel the cardiac developmental process and understand factors required for efficient in vitro primary cardiomyocytes proliferation, induced pluripotent stem cells (iPSCs) from DMD individuals provide a precious source of in vitro-derived DMD cardiomyocytes for the purpose of disease modeling and drug testing. These are more expandable and faced with less ethical issues compared to studies in animal.

Induced pluripotent stem cells are pluripotent cells obtained from cellular reprogramming of somatic cells though forced exogenous expression of pluripotency determining factors. Pluripotent stem cells can potentially give rise to an unlimited number any possible cell type in the adult body, including cardiomyocytes, while simultaneously replenishing their own pool. When somatic cells from a DMD individual are reprogrammed, the generated iPSCs may be expanded greatly while still carrying the DMD mutation present in the original cell and are called DMD-iPSCs. Likewise, so do the cardiomyocytes produced from their differentiation, and are called DMD-Cardiomyocytes. This study aimed to set the protocol in our laboratory for the in vitro generation of DMD-cardiomyocytes from patient-specific DMD-iPSCs of a Pakistani patient for future research work in Pakistan on DMD disease modeling and drug testing for efficacy and toxicity.

Thus, the first objective of this study was DMD-iPSC differentiation towards DMD-Cardiomyocytes and the second was molecular characterization of the differentiated cells using Immunostaining to check for cardiac marker expression.

## METHODS

This experimental study involving human cell culture was carried out from December 2018 to January 2019 at Stem Cell Research and Regenerative Medicine Lab (SCRML) at Dow Research Institute of Biotechnology and Biomedical Sciences (DRIBBS), Dow University of Health Sciences (DUHS) in Karachi city of Pakistan. Ethical approval was obtained from DUHS’s Institutional Review Board (IRB-773/DUHD/Approval/2016/299).

For this study, we obtained DMD-iPSCs generated previously in our laboratory from cellular reprogramming of Urine Derived Cells (UDCs) isolated from the urine of a Pakistani DMD patient. Non-random purposive method had been employed for subject selection for urine collection at DUHS’s affiliated clinics; recruiting nine years old male DMD individual, whose diagnosis had been confirmed by neurologists based on his history, clinical findings, muscle biopsy result, serum creatine phosphokinase level, nerve conduction velocity and electromyography findings. Genetic testing had not been done but was part of another study in our laboratory.

### Inclusion & Exclusion Criteria

The inclusion criteria included the male gender and an age of 5-25 years, besides a pre-diagnosed DMD status. The exclusion criteria included known urinary tract tumors, acute-renal failure and dialysis. Informed written consent was obtained from the participant and his guardian.

All work involving cell culture including reagent preparation was performed in a clean room with HEPA-filtered air, inside a class II biosafety cabinet (Thermo Fisher, 1300 Series A2).

First, DMD-iPSCs were plated in a well of a 12 well plate on Geltrex matrix (Gibco, cat#A1569601) at 10µg/cm^2^ in 1ml/well complete Essential 8 medium (Gibco, cat#A1517001) and incubated in a humidified incubator at 37°C and 5% CO_2_ (Eppendorf, Galaxy 170R). The iPSCs underwent daily medium change and were expanded to obtain an optimal confluence of 35-60% for differentiation. The PSC Cardiomyocyte Differentiation Kit (Gibco, cat#A2921201) consisting of Cardiomyocyte Differentiation Medium A, Cardiomyocyte Differentiation Medium B and Cardiomyocyte Maintenance Medium was used according to the manufacturer’s guidelines for the differentiation of iPSCs. Medium was changed very gently on alternate days. On day0 of differentiation, the medium was changed to 1ml/well Cardiomyocyte Differentiation Medium A, followed by 1ml/well Cardiomyocyte Differentiation Medium B on day 2, and 1ml/well Cardiomyocyte Maintenance Medium on day 4, day 6, day 8 and day 10. Twice, cells from a single UDC line were reprogrammed and differentiated into cardiomyocytes. Thus, differentiation was repeated twice on n = 1 patient.

The differentiated cells were checked for the expression of cardiomyocyte markers on day 11 of differentiation using Immunostaining with the Human Cardiomyocyte Immunocytochemistry Kit (Invitrogen, cat#A25973). The well was fixed for 15 minutes using 0.8ml 4% Formaldehyde in DPBS (Dulbecco’s Phosphate Buffered Saline), permeabilized using 1% Saponin in DPBS and blocked using 3% Bovine Serum Albumin in DPBS. Incubation with primary antibody diluted to 1× in blocking solution was done for three hours at room temperature followed by secondary antibody diluted to 1× in blocking solution for one hour at room temperature. Expression of the cardiomyocyte marker TNNT2 (gene for cardiac Troponin T) was checked using Mouse Anti-TNNT2 Antibody as primary antibody and Donkey Anti-Mouse Alexa Fluor 488 Antibody as secondary antibody. Expression of the cardiomyocyte marker NKX2-5 was checked using Rabbit Anti-NKX2-5 Antibody with Donkey Anti-Rabbit Alexa Fluor 594 Antibody. DAPI (4’ 6-Diamidino-2-phenylindole) was used for nuclear staining.

## RESULTS

**Fig.1:**
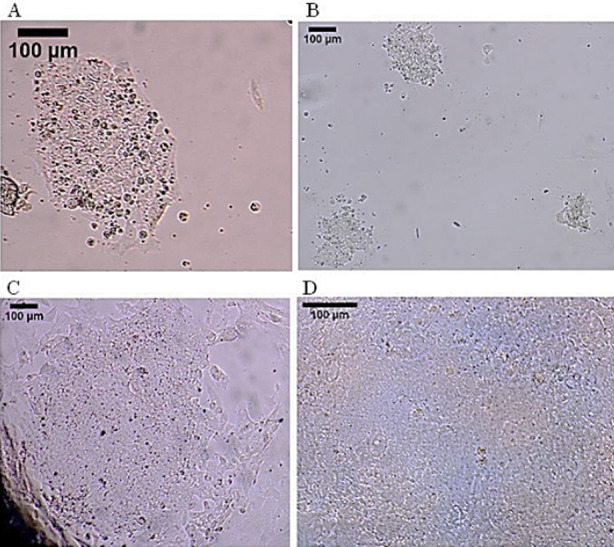
Induced pluripotent stem cell culture: (A-C) Colonies of iPSCs; (D) Morphology of iPSCs in colony at high power; seen as a homogenous population of spherical cells with large nuclei and high nuclear-cytoplasmic ratio. (Scale bar = 100µm).

The morphology of patient specific DMD-iPSCs used was observed to comprise of colonies with uniform margins consisting of a relatively homogenous population of spherical cells with large nuclei (high nuclear cytoplasmic ratio) as seen in Fig.1.

**Fig.2:**
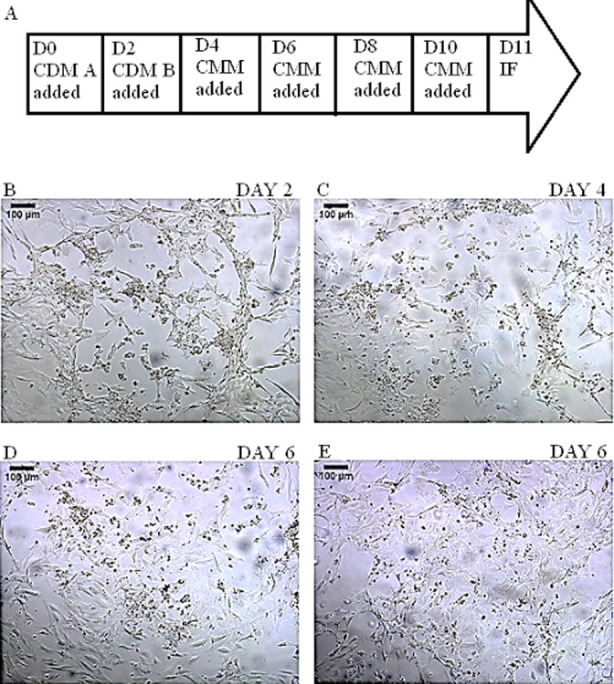
Differentiation of DMD-iPSCs into DMD-cardiomyocytes: (A) Schematic diagram summarising the workflow; (B-E) Changing cellular morphology along with significant cell death and shedding as seen on differentiation Day 2, 4, 6 and 6 respectively. (Scale bar = 100µm). Key: CDM: Cardiomyocyte Differentiation Medium; CMM: Cardiomyocyte Maintenance Medium; IF: Immunofluorescence.

Subsequent changes in cellular morphology were observed during the differentiation process. There was a lot of cell death and shedding and the cells appeared opaquer. The left-over differentiated cells lost the typical round, tightly packed iPSC morphology and appeared to be irregularly shaped larger cell bodies with cytoplasmic extension merging into each other with no obvious cell margins in between them (Fig.2).

**Fig.3:**
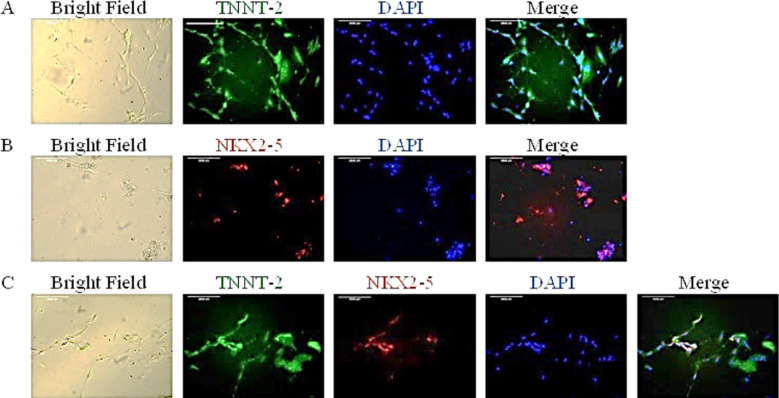
Immunofluorescence of the differentiated cells on day 11 of differentiation for the cardiomyocyte markers TNNT-2 and NKX2-5: From left to right, bright-field image, immunofluorescence image for the cardiomyocyte marker, blue immunofluorescence image for the nuclear-counter stain DAPI and the merged image for the cardiomyocyte marker with DAPI: (A) Green immunofluorescence for TNNT2 (20×); (B) Red immunofluorescence for NKX2-5 (20×); (C) Green immunofluorescence for TNNT2 and red immunofluorescence for NKX2-5 (20×). (Key: DAPI, 4’ 6-Diamidino-2-phenylindole; NKX2-5, NK2 home box 5; TNNT-2, Gene for cardiac Troponin T)

Molecular characterization of the differentiated cells on day 11 of differentiation demonstrated positive immunofluorescence for the expression of the cardiomyocyte markers TNNT-2 and NKX2-5. Green immunofluorescence was observed for TNNT-2 while red immunofluorescence was observed for NKX2-5 (Fig.3).

## DISCUSSION

In the past studies, in vitro generation of cardiomyocytes from pluripotent stem cells through spontaneous differentiation in embryoid bodies (aggregates of pluripotent stem cells in suspension), culture with cardio-inductive cells like endoderm-like cell line (END-2) derived from murine embryonal carcinoma, or in media conditioned by them have shown poor efficiency.^12^

Endeavors for unraveling the complex pathways involved in vivo in cardio myogenesis have revealed temporal expression and regulation of transcription factors, mRNAs and growth factors.^13^ Fibroblast Growth Factor (FGF), Bone Morphogenetic Protein (BMP)/ Transforming Growth Factor β (TGFβ) and Wingless-related mouse mammary tumor virus integration site (Wnt) are the three major pathways recognized as regulators of mesoderm formation and subsequently cardiomyogenesis from it in addition to microRNAs including miR-1 and miR-133.^14^ This knowledge about the in vivo process has been used to designs in vitro protocols for the differentiation of iPSCs towards cardiomyocytes. A popular approach to achieve cardiac differentiation in vitro is the introduction of small molecules in a time and concentration sensitive manner in order to activate and then sequentially inhibit the Wnt pathway as demonstrated by Lian et al. and Sharma at al.^15^,^16^ Wnt is activated by Glycogen Synthase Kinase-3β inhibitors like CHIR99021 that prevent its degradation, and Wnt is inhibited by PORCN inhibitors like IWP2 and Wnt-C59 that prevent its production.

To the best of our knowledge, this is the first time iPSC-derived DMD-Cardiomyocytes have been generated for a Pakistani patient in vitro and no local studies have been published previously on In vitro DMD-cardiomyocytes of Pakistani patients from any source. Very few studies have investigating DMD mutation in Pakistan. Hassan et al. in 2008 found that consistent with geographic variation in DMD mutations, intra-genic deletions were lower in Pakistan (40.75%), compared to the generally reported level (65%), which was similar to other Asian nations.^17^ Ansar et al. in 2019 found deletions in exon 42-52 (exon 46 most frequent) and duplications in exon 3-7 to be the most frequent in Pakistani patients; while global data reveals exon 45 as the most frequent deletion but exon 2 as the most frequent duplication.^18^

Urine is a suitable non-invasive, painless and convenient source of DMD patients’ cells for generating DMD-iPSCs.^19^ Hence, in this study, patient-specific urine-derived DMD-iPSCs have been chosen as starting material and been differentiated towards patient-specific DMD-Cardiomyocytes, as has been demonstrated previously by others like Guan et al. and Pioner et al.^20^,^21^ In this study, we have used Gibco’s PSC Cardiomyocyte Differentiation Kit. Although knowledge about its constituents is proprietary information, it is intended to simplify the work-flow and be more economical than several expensive small molecules purchased individually, as also demonstrated previously by Virtanen et al. and Robert et al.^22^,^23^

In literature, cardiomyocytes have been reported as expressive of the cardiac markers TNNT-2 and NKX2-5, as well as α-actinin, MLC2a (myosin light chain 2a), MLC2v (myosin light chain 2v) and MYH6 (myosin heavy chain).^24^,^25^ In this study, identity of the differentiated cells generated has been confirmed as cardiomyocyte by showing expression of the cardiac markers TNNT-2 and NKX2-5.

### Limitations of the study

Functional assays for the invitro derived cardiomyocytes including electrophysiological analysis using patch clamp and analysis of mechanical beating behavior were not done due to time and funding limitations.

## CONCLUSION

The non-invasively, painlessly and conveniently sourced urine-derived iPSCs from Pakistani DMD patients can be used to generate patient-specific DMD-Cardiomyocytes using well defined growth media and factors through this simple and economical protocol; for application in drug-testing for tailored personalized therapy, disease modeling, new drug testing for efficacy and toxicity.

### Authors Contribution:

**FFG** conducted all the experiments under supervision and wrote the manuscript. **MW** designed the study, supervised all the experiments, and performed review and final approval of manuscript
